# Rehabilitation and Disability Spectrum From Adverse Childhood Experience: The Impact of the Movement Cognition and Narration of Emotions Treatment (MCNT) Version 2.0

**DOI:** 10.3389/fpsyt.2020.609819

**Published:** 2021-01-25

**Authors:** Gisella Baglio, Michela Zanette, Monica Di Cesare, Sonia Di Tella, Mario Clerici, Francesca Baglio, Valeria Blasi

**Affiliations:** ^1^Istituto di Ricovero e Cura a Carattere Scientifico (IRCCS), Don Carlo Gnocchi Foundation Onlus, Milan, Italy; ^2^Department of Pathophysiology and Transplantation, University of Milan, Milan, Italy

**Keywords:** adverse childhood experience, cognitive-behavioral and motor impairment, borderline intellectual functioning, multimodal rehabilitation, stressful environment, emotional deregulation

## Abstract

Adverse Childhood Experiences (ACE) are associated with an increased risk of cerebral, behavioral, and cognitive outcomes, and vulnerability to develop a Borderline Intellectual Functioning (BIF). BIF is characterized by an intelligence quotient (IQ) in the range 70–85, poor executive functioning, difficulties in emotion processing, and motor competencies. All these difficulties can lead to mental and/or neurodevelopmental disorders that require long-term care. Accordingly, we developed an intensive and multidomain rehabilitation program for children with ACE and BIF, termed the Movement Cognition and Narration of emotions Treatment (MCNT1.0). The efficacy of MCNT1.0 on cognitive and social functioning was demonstrated with a previously reported randomized controlled trial (RCT). To extend the impact of the treatment also to the motor domain a new version, called MCNT2.0, was implemented. The present study aims to verify the feasibility of MCNT2.0 and its effects on the motor domain. A quasi-experimental approach was used in which a group of 18 children with ACE and BIF were consecutively recruited and participated in the MCNT 2.0 program. Participants were compared with the MCNT1.0 group as an active comparator, using the dataset of the RCT. The two groups received a full evaluation comprising: the Wechsler Intelligent Scale for Children-IV (WISC-IV), the Movement-ABC (M-ABC), the Test of Gross Motor Development (TGMD), the Social Skills from Vineland Adaptive Behavioral Scale-II (VABS-II) and the Child Behavior Check List 6–18 (CBCL). An ANCOVA was carried out on changes in the scale scores from baseline with age and baseline score as covariates. Results showed a mean adherence to treatment of 0.85 (*sd* = 0.07), with no differences between groups in IQ, and Social Skills changes, while greater improvements for motor abilities were shown in the MCNT 2.0 group: M-ABC (*p* = 0.002), and TGMD (*p* = 0.002). Finally, greater improvement in the CBCL scale was observed in the MCNT 1.0 group (*p* = 0.002). Results indicate that due to its positive effects on cognitive, social participation and motor domains, MCNT2.0 may represent a protective factor against maladaptive outcomes of children with ACE and BIF.

## Introduction

Adverse environmental conditions are frequently associated with neuropsychiatric consequences during early age. Some studies suggested how the exposure to adverse childhood experience (ACE) contributes to altered structure and function in several neurobiological systems, mostly related to the limbic system, with a consequent “latent vulnerability” to multiple forms of youth and/or adult psychopathology (1–3). ACE can be defined as experiences requiring “significant adaptation by an average child” (4) and include “harms that affect children directly (e.g., abuse and neglect) and indirectly through their living environments (e.g., parental conflict, substance abuse, or mental illness)” (5). Borderline intellectual functioning (BIF) is an important and frequently unrecognized comorbid condition (6–9) with an increased risk of exposure to ACE compared to their peers (10) such as inadequate housing, low parental education, low social class, low income, absence of a parent, parental psychiatric morbidity (10). BIF is defined as a boundary condition between typical development and intellectual disability, characterized by an intelligence quotient (IQ) within the range 70–85, associated with difficulties in social participation and adaptability, and is recognized as a V code in the Diagnostic Statistical Manual-5 (11). Children with BIF exhibit difficulties in several developmental domains such as cognition, affectivity, sociality, and movement: learning disorders, impairment in executive functioning, receptive and expressive language, motor planning, emotional regulation, Theory Of Mind and behavioral difficulties are often detected (7, 12–18). Interestingly, children with BIF also exhibit a peculiar pattern of sleep organization characterized by an alteration of the cyclic alternating pattern, and a positive correlation between sleep duration and intellectual abilities (19, 20). In line with such evidence, several studies found a significant correlation between ACE and sleep disorders (21).

It has been shown that deprived environments negatively impact working memory, inhibitory control, cognitive flexibility (22–27), language (28, 29), and global intelligence (30). Accordingly, a recent study of a large cohort of 14,000 children showed that by the age of 2 years, children belonging to a low socio-economic environment had a 6 point lower IQ compared to their high socio-economic peers. This difference almost tripled when the same subjects were evaluated at the age of 16 (31). Finally, unpredictable and potentially threatening environments negatively impact the development of the emotional response and regulation systems with consequences on behavior and social relationships (4).

Children with ACE and BIF are thus a highly vulnerable population at risk of maladaptive outcomes, such as lifetime cognitive and mental disorders, anxiety, depression, substance abuse, externalizing/internalizing behavioral disorders, drop-out from schooling, and low income if left untreated.

To prevent the psychopathological drift following ACE and to respond to the several special needs of these children, we developed a rehabilitative intervention, termed the Movement Cognition and Narration of emotions Treatment [MCNT; (32)], which aimed at improving global intelligence, movement abilities and adaptive competences. MCNT lasts 9 months and consists in 3 h a day, 5 days a week of 3 laboratories: the Movement Lab, to improve fine and gross motor abilities; the Cognitive Lab, to improve reasoning, mental flexibility and wider executive functioning; and the Emotion Lab, to improve emotion recognition, comprehension and expression [for a detailed description of MCNT see the study protocol published (32)]. Indeed, this method targets the motor, cognitive and affective domains.

The efficacy of this intensive and multidomain experimental approach was investigated with a randomized controlled trial [RCT; (33)]. Results demonstrated that MCNT, which we shall rename here MCNT 1.0, was more effective than Standard Speech therapy (SST, usual care) in improving intellectual, adaptive and behavioral functioning in children with ACE and BIF, whereas no significant improvement was observed in motor abilities.

Moving from our previous results, we have focused on an *ad hoc* adjustment of the MCNT method, modifying the Movement Lab. We shall rename this new version as MCNT 2.0, in which we have abandoned the game therapy approach in the Movement Lab in lieu of a method focused on movement and body awareness, the Body Minding.

In the light of our previous results, which showed a poor effect of SST on cognitive and adaptive functioning, we preferred not to carry out a RCT with the SST as a control group but to directly compare the two versions of the MCNT intervention. Indeed, a treatment with poor efficacy might prevent the gain of competences relevant for children's development. Moreover, RCTs are expensive and not easy to implement in a routine care setting. For this reasons, we designed a quasi-experimental study (34) in which a group of 18 children with ACE and BIF were consecutively recruited to participate in the MCNT 2.0 program. This group was then compared with the children treated with MCNT 1.0 from the original RCT [(32, 33)].

The aims of the current study were to evaluate the feasibility of MCNT 2.0 intervention and its effect on motor skills in a sample of children with ACE and BIF. Based on our previous experience, we expected MCNT 2.0 to be feasible, and that compared to MCNT 1.0, the newer implementation would reveal a significant positive effect on motor abilities, without any differences between the two interventions on cognitive, social, and behavioral competencies.

## Materials and Methods

### Study Design and Participants

The Study was approved by the Ethics Committee of the Don Gnocchi Foundation (DGF) and of the ASST S. Paolo and S. Carlo Hospital. All parents signed a written informed consent at the first meeting.

This was a quasi-experimental study in which a group of children with BIF and exposed to ACE were consecutively recruited and treated with MCNT 2.0, with all the children belonging to the group treated with MCNT 1.0 as an active comparator, consisting of 18 subjects that underwent the rehabilitation intervention in the Years 2016–2017. The MCNT 1.0 group belongs to the dataset of our previous RCT (33).

Participant assignment in the MCNT 2.0 group was not randomized. However, to support internal validity and to reduce sample differences, participants were selected from the same catchment area of the city of Milan and with the same inclusion/exclusion criteria of our previous RCT.

Inclusion criteria were: age range between 6–11 years old and attending primary mainstream school; a Full Scale Intelligence Quotient (FSIQ) score ranging from 70 to 85; presence of an impact on social functioning (school and/or family context) as derived from the clinical history. Since all children participating in the MCNT 1.0 program belonged to a middle, middle-low or low socio-economic status [SES < 39, (35)], we wanted the two groups to be matched for this parameter to control for confounding variables that may threaten the internal validity of the study. For this reason, we verified that none of the children of the MCNT 2.0 were of a high SES background.

Exclusion criteria were: presence of major neuropsychiatric disorders (such as ADHD and autism spectrum disorder); presence of neurological conditions such as epilepsy, traumatic brain injury, brain malformation and infectious disease involving the central nervous system. Other exclusion criteria considered were: the presence of systemic diseases such as diabetes or dysimmune disorders, genetic syndromes such as Down syndrome or Fragile X syndrome. Furthermore, a positive history for psychoactive drugs, particularly referring to current or past use of psychostimulants, neuroleptics, antidepressants, benzodiazepines, and antiepileptic drugs were also considered exclusion criteria.

The MCNT 2.0 group consisted of 18 children with ACE associated with BIF (age: mean = 7.68; *sd* = 1.25) and attending mainstream primary school in Italy treated with MCNT 2.0 (see methods section) in the years 2018/2019. Initially, 19 participants were enrolled but one child abandoned the study and was excluded from analyses. The MCNT 1.0 group consisted of 18 children with ACE and BIF (mean age = 7.78; *sd* = 1.31).

Characteristics of two groups at baseline are presented in Table 1.

**Table 1:** Demographic and baseline data.

**Variable**	**MCNT^**1.0**^**	**MCNT^**2.0**^**	**Group comparison (*p*-value)**
Subjects (Number)	18	18	
Sex (M:F)	8:10	7:11	1.0
Age (Years, Mean ± *SD*)	7.78 ± 1.31	7.67 ± 1.28	0.83
SES^*^ (Mean ± *SD*)	24.03 ± 11.64	21.69 ± 9.95	0.34
ESCL^*^ (Mean ± *SD*)	3.67 ± 2.85	4.22 ± 3.14	0.55
WISC-FSIQ (Mean ± *SD*)	75.11 ± 8.52	77.22 ± 5.33	0.84
Adherence to treatment	0.88 ± 0.07	0.84 ± 0.07	0.09

*MCNT, Movement Cognition and Narration of the emotions Treatment 1.0 and 2.0 version; SES, Socio-Economic Status; ESCL, Environmental Stress Checklist; WISC, Wechsler Intelligent Scale for Children; FSIQ, Full Scale IQ*;

* *the variable was not normally distributed and thus the Mann-Whitney test was used*.

### Clinical Assessment on ACE, Motor, Cognitive, and Behavioral Domains

To detect the presence of ACE we used the Environmental Stress Check-List [ESCL; (2)]. The ESCL consists in a listing of the V-codes from DSM-5, and Z-codes from ICD-10, that explore problems related to relational, neglect, physical, sexual and/or psychological abuse, educational and occupational, housing and economic, social exclusion or rejection, plus the presence of social services intervention, of major psychiatric diagnosis, and of substance abuse within the family members. A 0 (absence) to 1 (presence) score was attributed to each item after careful consideration of its relevance for the clinical manifestations. The ESCL total score ranges from 0 to 24 with higher values indicating a greater number of environmental stressful conditions.

The clinical assessment on motor, cognitive and behavioral domains was carried out at two time points (T0, within 2 months prior to the beginning of the treatment and after 9 months at T1 within 2 months after the end of the treatment).

Motor domain was assessed by a Neuro-Psychomotor in Developmental Age Therapist using:

1. The Movement-ABC [M-ABC; (36)], for the assessment of the motor skills, included manual dexterity, ball skills and static/dynamic balance; the total score was expressed in percentiles and fall into clinical range for scores below 6th percentile, into borderline range from 6th to 15th percentile and into normal range above 15th percentile;
2. The Test of Gross Motor Development [TGMD; (37)] through the assessment of both Locomotor and Object Control abilities give a measure of gross motor skill development, the Gross Motor Quotient (GMQ);

Cognitive domain was assessed by a neuropsychologist using:

1. The Wechsler Intelligence Scale for Children-IV [WISC-IV; (38)] to measure global intellectual functioning as full scale IQ (FSIQ) and the following indices: verbal comprehension index (VCI); perceptual reasoning index (PRI); working memory index (WMI); processing speed index (PSI). Children belonging to the MCNT 1.0 group were evaluated with the previous version of the scale, the WISC-III (39). Despite substantial differences between the two versions in the structure (the later revision of the Scale removed three and introduced five new subtests modifying its structure) and the construct of the indices and of the FSIQ, there is a very high correlation (0.89) in the FSIQ between the two versions, as previously reported (40). Indeed, we decided to use the FSIQ for the between group comparison because it is more stable and reliable when compared to the indices scores across the two versions of the WISC scale. Moreover, to avoid direct comparison of the scores of the two versions, we used delta values (post-pre-treatment scores), a longitudinal single subject change over time approach, to compare the two groups of children.

Behavioral and social skills were assessed by a psychologist using:

1. The Vineland Adaptive Behavioral Scale II [VABS-II; (41)], to assess social functioning, communication abilities, daily living skills, and a full scale quotient (FSQ) of adaptive functioning through a single interview with parents.
2. The Child Behavior Checklist 6–18 [CBCL 6–18; (42, 43)], in the Parent's Report Form, to evaluate emotional and behavioral problems in children and adolescents; data were expressed in Tscore and higher scores indicate greater problems; the total score can be interpreted as falling in the normal (<60 Tscore), borderline (60–63 Tscore), or clinical range (>63 Tscore).

### Intervention: the MCNT 2.0

The MCNT2.0 is an adapted version of MCNT1.0, an intensive and multimodal rehabilitation program, whose effectiveness has been demonstrated in our previous study (33). A detailed description of this method is present in the study protocol (32). MCNT consists of three laboratories (Lab), the Movement Lab, the Cognitive Lab and the Emotion Lab, in which children work in small group of seven to eight. Due to the lack of significant results, the Movement Lab was the only one that underwent substantial adjustment from MCNT 1.0–2.0.

Briefly, the first laboratory, the Cognitive Lab, aims at cognitive empowerment working on executive skills, such as fluid reasoning, problem solving, attention, inhibitory control, monitoring, switching, and academic competencies (reading, writing, and calculating) including listening comprehension with the use of the multimedia interactive whiteboard (MIW). The second lab, the Emotion Lab, adopts a relational dynamic approach to improve emotion expression, recognition, comprehension, and autoregulation. Purpose of the Emotion Lab is the “alphabetization” of the emotions (44). Spontaneous play, drawing, stories (invented and/or dramatized) and talking are the preferred tools used by the psychotherapist to achieve these goals. Finally, the third laboratory, the Movement Lab, fostered the improvement of global motor functioning with an game therapy approach using commercial gaming consoles. In the MCNT 2.0 the game therapy was substituted with a method focused on movement and body awareness. The focus was the body as a means through which we move in space and experience feelings. In more details, the Movement Lab 2.0 included two types of activities: (1) bodily movements to work on the “acting self” (45, 46) that is the sense of agency of own body during an action and include feelings and movement controls. This activity included sequences of coordination movements, from the simplest to the most complex, that involved bimanual and interlimbic coordination patterns but also eyes, ears, tongue, breath, and other cross movement designed for stimulating hemispheric brain interconnection and improving balance, stability, coordination and planning, speed, and accuracy in the movements. During the performance, children's attention was usually driven on “feeling the body.” Common games such as jumping rope, hopscotch, target shooting, balance play were performed to increase coordination, speed and accuracy; and (2) bodily awareness/perception to work on the “sensorial self” that is the sense of ownership of body and perceptual experience of all body parts in the external space (45–47). The child was guided toward the representation of his/her body through relaxation techniques guided by imaginative processes.

The MCNT 2.0 treatment was carried out for 9 months in a hospital setting. Children were accompanied from school to our Center with a shuttle service provided by our Institution. Children attended all the three Labs every day for 5 days/week, 3 h/day in the afternoon, working in small groups. For each group two specialized operators were assigned. Moreover, weekly meetings among professionals were carried out to monitor treatment, and to discuss emerging difficulties. Finally, at least two meeting with teachers, and at least 5 meetings with children's parents were provided. The number of the meetings was based on the specificity of each child situation. These meetings had the objective to create a support network and discuss the methodology of the intervention, evaluate the specific needs of each child, and find solutions to problems as they arose.

Adherence to treatment for each participant was calculated as the number of attended sessions divided by the number of total sessions and used as a measure of feasibility.

### Statistical Analysis and Sample Size Calculation

Since no preliminary data relative to motor abilities from our lab were available, the sample size was calculated according to data from the literature (48). The *a priori* sample size calculation was performed with G^*^Power software 3.1, considering a medium effect size (Cohen's *f*) = 0.25 (49), with an expected power of at least 0.80 and an alpha value 0.05. According to this procedure the estimated sample size *a priori* was 34.

Statistical analysis was conducted using SPSS software (version 24). Before proceeding to hypothesis testing, we checked the normal distribution for all measures using both the Kolmogorov-Smirnov and Shapiro-Wilk tests. A parametric (one-way ANOVA) or non-parametric (Mann-Whitney) comparison was performed as appropriate to compare baseline demographic and clinical characteristics of the two groups of children, MCNT 1.0 and MCNT 2.0. A chi-squared was used to test differences between groups for sex.

The measures considered in this study were changes in the scale scores from baseline (delta scores). For variables not normally distributed, a Bloom's transformation was applied to normalize scores. An analysis of covariance (ANCOVA), with age and pre intervention evaluation score as covariates, was carried out. Score differences were described using estimated mean, mean difference, and *R*^2^ model fitting. An α value of 0.05 was considered statistically significant, and all comparisons were 2-tailed. The false discovery rate (FDR) correction was used to adjust for multiple comparisons between the different measures (50).

The magnitude of effects was calculated and reported with effects size η^2^ interpreted as follows: 0.01 as a small effect; 0.06 as an intermediate effect; 0.140 and higher as a strong effect (51).

Except for M-ABC and CBCL all data were expressed in standard score.

Data relating to the Communication scale, Daily Living Skills and FSQ of VABS-II and the four indices of WISC-IV were investigated with a paired *t*-test in the MCNT 2.0 group only.

## Results

### Demographics and Feasibility

Table 1 shows baseline comparison between MCNT 1.0 and 2.0 groups. The two groups were matched for sample size, age, sex, SES, ESCL, and FSIQ.

Adherence for MCNT 1.0 was 0.88 (*sd* = 0.07), for MCNT 2.0 was 0.84 (*sd* = 0.07) with no statistical difference (Table 1). Both groups showed a very small number of drop-outs: 2 children in the MCNT 1.0 (one child moved to another country before post treatment evaluation, while another child had difficulties in working in a group setting) and 1 child in the MCNT 2.0 group (home to hospital distance was too great for the family to manage).

A detailed listing of the prevalence of each environmental stressor for each group was illustrated in Table 2.

**Table 2:** Environmental Stress Check List scoring.

	**MCNT 1.0 *N* of subjects**	**MCNT 2.0 *N*. of subjects**
V60.1 (Z59.1)—Inadequate Housing	3	2
V60.2 (Z59.6)—Low income	10	5
V61.03 (Z63.5)—Disruption of family by separation or divorce	2	5
V61.20 (Z62.820)–Parent-child relational problem	3	7
V61.21 (Z69.010)—Encounter for mental health services for victim of parental child physical abuse	2	3
V61.21 (Z69.020)—Encounter for mental health services for victim of non-parental child physical abuse	0	0
V61.21 (Z69.010)—Encounter for mental health services for victim of parental child sexual abuse	0	0
V61.21 (Z69.020)—Encounter for mental health services for victim of non-parental child sexual abuse	2	0
V61.21 (Z69.010)—Encounter for mental health services for victim of parental child neglect	4	0
V61.21 (Z69.020)—Encounter for mental health services for victim of non-parental child neglect	0	0
V61.21 (Z69.010)—Encounter for mental health services for victim of parental child psychological abuse	0	3
V61.21 (Z69.020)—Encounter for mental health services for victim of non-parental child psychological abuse	0	2
V61.29 (Z62.898)—Child affected by parental relationship distress	2	2
1.V61.8 (Z62.891)—Sibling Relational problem	0	1
V61.8 (Z62.29)—Upbringing away from parents	0	2
V61.8 (Z63.8)—High expressed emotion level within family	6	2
V62.3 (Z55.9)—Academic or educational problem (Underachievement in school)	17	18
V62.3 (Z55.9)—Academic or educational problem (School-Family conflicts)	1	5
V62.4 (Z60.3)—Acculturation difficulty	4	5
V62.4 (Z60.4)—Social exclusion or rejection	2	1
(Z63.2)—Inadequate family support	3	5
(Z63.3) Absence of family member	0	4
Social Services Intervention	4	2
Major Psychiatric Diagnosis within the family	1	0
Substance abuse within the family	0	2

*Prevalence of Environmental Stress factors in the group MCNT 1.0 and MCNT 2.0*.

### Between Group Comparison

To determine the effects of MCNT 2.0 on motor, cognitive and behavioral/social competencies, an ANCOVA analysis was performed. Delta values (post-pre-treatment) of each scale were compared between groups with age and baseline evaluation as covariates.

Results are reported in Table 3. To summarize, the MCNT 2.0 group showed greater motor ability improvements compared to MCNT 1.0, as detected by both scales: M-ABC (*p* = 0.002, before Bloom's transformation delta values for MCNT 1.0 mean = 7.85, *sd* = 15.19; MCNT 2.0 group mean = 40.95, *sd* = 21.89), and TGMD (*p* = 0.002). For cognitive abilities detected with the FSIQ, and for Social Skills as detected by the VABS II scale, no significant differences were detected between the two groups. For behavioral competencies, detected with the CBCL scale, a greater improvement was observed in the MCNT 1.0 group (*p* = 0.002).

**Table 3:** ANCOVA analysis of changes from the baseline, with age and baseline score as covariates.

**Scale**	**MCNT 1.0 Estimated Mean (St. Err.)**	**MCNT 2.0 Estimated Mean (St. Err.)**	**MCNT 1.0 vs. MCNT 2.0 Mean Difference (St. Err.)**	***F***	***P*-value (FDR)**	**η^2^**	**ω**	**Adj. *R*^**2**^**
FSIQ	10.21 (2.56)	7.57 (2.56)	2.65 (3.64)	0.53	0.473	0.016	0.109	0.037
M-ABC^*^	−0.74 (0.23)	0.54 (0.19)	−1.27 (0.33)	15.28	**0.002**	0.361	0.965	0.381
TGMD	2.99 (2.55)	21.17 (2.39)	−18.18 (3.72)	23.95	**0.002**	0.444	0.997	0.422
CBCL	−10.32 (1.86)	2.19 (1.86)	−12.51 (2.66)	22.17	**0.002**	0.460	0.995	0.600
Social-VABS II	9.53 (2.44)	5.47 (2.22)	4.06 (3.50)	1.35	0.325	0.063	0.197	0.395

*MCNT, Movement Cognition and Narration of the emotions Treatment 1.0 and 2.0 version; St. Err, Standard Error; WISC III/IV, Wechsler Intelligent Scale for Children; FSIQ, Full Scale IQ; M-ABC, Movement Assessment Battery for Children (data expressed in percentiles)*;

* *due to non-normal distribution of this variable a Bloom's was transformation applied; TGMD, Test of Gross Motor Development; TGMD, Test of Gross Motor Development; VABS-II, Vineland Adaptive Behavior Scale II; FDR, False Discovery Rate correction; ω, power; η^2^, Partial Effect Size; Significant results are reported in bold font*.

### Within MCNT 2.0 Group Comparison

To better detail the changes in the intellectual and adaptive functioning of the MNCT 2.0 group only, a paired *t*-test was performed on pre vs. post treatment scores.

Results are reported in Table 4. Data showed significant difference between pre and post treatment scoring for Perceptual Reasoning Index (PRI, *p* = 0.04) within the WISC IV evaluation, and for Communication (*p* = 0.04), Daily Living Skills (*p* = 0.04), and the Full-Scale Quotient (*p* = 0.04) within the VABS II.

**Table 4:** Pairwise comparison pre vs. post-MCNT2.0 scores of WISC IV indices and VABS –II sub-scales.

	**Scale**	**Variables/Score**	**MCNT**^**2.0**^ **(*****N*** **=** **18)**		**Pairwise comparison T0 vs. T1**	**Cohen's *d***
			**T0 Mean (*SD*)**	**T1 Mean (*SD*)**	**FDR*-p-*value**	
Intellectual functioning	WISC-IV	VCI	82.11 (8.44)	86.44 (12.22)	0.29	0.40
		PRI	86.22 (9.33)	96.44 (15.90)	**0.040**	**0.74**
		WMI	78.33 (7.81)	82.33 (12.85)	0.22	0.36
		PSI	84.05 (11.42)	86.22 (13.92)	0.51	0.17
Adaptive functioning	VABS-II	FSQ	86.92 (10.18)	94.85 (12.13)	**0.040**	**0.70**
		Communication	81.08 (12.15)	90.38 (14.03)	**0.040**	**0.71**
		Daily living skills	90.38 (10.54)	98.92 (13.62)	**0.040**	**0.69**

*MCNT, Movement Cognition and Narration of the emotions Treatment; WISC IV, Wechsler Intelligent Scale for Children IV; VCI, Verbal Comprehension Index; PRI, Perceptual Reasoning Index; WMI, Working Memory Index; PSI, Processing Speed Index; VABS-II, Vineland Adaptive Behavioral Scale II; FSQ, Full Scale Quotient; FDR, False Discovery Rate correction; Ns, not significant; Significant results are reported in bold font*.

## Discussion

In this work we report data showing the feasibility in term of treatment adherence of the MCNT 2.0 and its greater effects on motor abilities compared to the previous version of the treatment, the MNCT 1.0.

The previous version of the MCNT treatment, MCNT 1.0, was investigated with an RCT study (33) whose results showed a positive effect of the treatment on intellectual, social and behavioral competences in comparison to standard care (i.e., individual speech therapy). Despite MCNT 1.0 having implemented interventions targeting motor competences, significant improvement was not detected in this regard. For this reason, an adjusted version of MCNT was created, in which the movement training component was modified with the introduction of the Body-Minding, a body awareness and interlimbic coordination program; this version was re-named MCNT 2.0. Accordingly, first aim of this study was to evaluate the feasibility and the effects of this modification on the motor competencies. To this purpose, we conducted a quasi-experimental study in which data relative to a non-randomized group of children was compared to a secondary dataset from the previously mentioned RCT (34, 52).

The first result is the feasibility of the MCNT 2.0. Despite the intensity and the long duration of the treatment, we observed a very high adherence coupled with a very low number of drop-outs, demonstrating the feasibility of the MCNT 2.0 program with no differences with the original treatment. In both versions, a shuttle service accompanied children from school to our Institution. Moreover, both versions of the MCNT provided support for the participant's teachers and their families. Having facilitated the access to our Institution and having provided support to the schools enabled the creation of a network supporting children and their families that we believe explains such a high adherence to the rehabilitation treatment.

Another important result of the present study is the confirmation that the MCNT method is effective in the improvement of the intellectual abilities of children with BIF. This is in line with the results of the previous RCT study (33). Specifically, no difference between the two groups was observed in the changes observed after treatment. The two groups showed similar improvement in the full-scale IQ. Moreover, data from the MCNT 2.0 group, showed that this datum was likely due to the increment in the Perceptual Reasoning, as shown by the increment in the PRI. These data are in agreement with the results of our previous study (33) in which an increment in the Performance Quotient was observed in the MCNT 1.0 group. Both indices are measures of Fluid Reasoning (Gf), Comprehension-Knowledge (Gc), and Visual Processing (Gv). The metacognitive strategies used into the Cognitive Lab, which remained unmodified compared to the previous version, facilitated the empowerment of creative thinking through the exploration of new solutions, different perspectives, brainstorming techniques, and semantic association. As a consequence, the participants were able to create conceptual links, improved their long-term memory, and were able to more finely monitor their own cognitive and decision-making processes. This result cannot be imputed to the training of specific abilities that are tested within the IQ evaluation. Indeed, the MCNT approach does not involve any type of targeted cognitive training. The latter approach is controversial because some authors claim that it is effective in improving only the specific cognitive component that is trained. In this respect, some studies highlighted the difficulties in generalizing the trained ability to new learning context, from experimental setting to real life for example (53). In the MCNT, the Cognitive Lab focused on metacognitive strategies that are transversal to several cognitive competences, and more ecological. Our data are relevant in terms of clinical prognosis, because the improvement of global intellectual functioning can have a positive effect also on adaptive skills. Accordingly, a recent study (54) showed poorer emotion processing in adolescents with BIF when compared to healthy controls, and an inverse relationship between intellectual functioning and emotional awareness. The authors interpreted their results as evidence that borderline cognitive functioning affects mentalization processes and thus adaptive skills. For this reason, we consider it important, when working with children with BIF and ACE, to focus the treatment also on the intellectual abilities as defined above.

The main result of the present study is related to the effects of MCNT 2.0 on motor competencies in children with ACE and BIF. Data presented showed a significant increase in fine and gross motor functioning, as assessed with M-ABC and TGMD in children that underwent MCNT 2.0. This result relates to the only difference between the two versions of the MCNT. In the first version of the intervention, a Game Therapy approach was used, based on the use of the Wii and Xbox video game platforms, while the present version applied the Body-Minding, a method focused on both body movements with bimanual and interlimbic coordination exercises and body awareness. The choice of a Game therapy approach in the MCNT 1.0 was based on previous research that demonstrated its good efficacy in promoting engagement, motivation and motor competence in children (55–58) also in case of mental disability (59) and hand-eye coordination in adults (60). The present data, though, showed greater efficacy of the Body-Minding approach. A possible interpretation of this datum is that despite the “Game Therapy” approach is highly motivating and engaging for children, it only allows the choice of the type of game and its level of difficulty but not a finer tuning of the activities due to platform/game limitations. Conversely, the Body-Minding approach, even in a group setting, fostered an intervention on motor coordination and planning, and proprioceptive feeling, that was personalized and tailored on the basis of each child's strengths and difficulties. These data are in agreement with a previous study by Ferguson et al. (48) in which a Nintendo Wii Fit Training was compared to a Neuromotor Task Training (NTT), both carried out in a group setting to evaluate the impact on the performance of children with motor coordination problems. Children that participated in this study, similarly to our sample, attended mainstream primary schools and came from a low-income environment. Results showed that the NTT approach achieved broader and greater success than Wii training in motor proficiency, cardiorespiratory fitness and functional strength. On the contrary, children that underwent the Wii training, improved their anaerobic performance but their motor performance remained within the at-risk range. These data are relevant in light of the poor motor competences typically shown by children with BIF and ACE due to their difficulties in locomotor, object control, and fine motor skills (17, 61, 62).

The herein results on the motor performance of children with ACE and BIF are relevant because the motor difficulties observed in these children are strictly linked with the cognitive processes involving executive functions, such as inhibitory control and planning (63). Moreover, fine motor skills are highly related to the possibility to improve cognitive skills in pre-school children with intellectual disability and learning disorders (64). Indeed, the information arising from within the body, through visual, auditory, olfactive, tactile, and proprioceptive pathway need to be rapidly and efficiently processed and integrated to achieve a body awareness and to produce a goal-directed movement. Thus, intervening on the motor domain, can have an effect also on executive functions and vice-versa. Finally, driving attention on bodily sensation helps to feel, recognize, discriminate and regulate emotions that would otherwise remain confused, unwanted, and unexpressed. Indeed, in addition to cognitive and motor difficulties, children with ACE and BIF show emotional and behavioral difficulties associated (7, 13, 14, 65) with deficits in social competencies (12). Both versions of MCNT rehabilitation program provided specific interventions for emotion narration and recognition, the Emotion Lab, whose efficacy in improving social functioning and behavior was shown by the results of the RCT study (33). The effect of treatment on social functioning was also confirmed in the MCNT 2.0 group. Moreover, significant increment in all indices of the VABS II scale was observed within this group. Unfortunately, the complete profile at the VABS II was available only for the MCNT 2.0 group and thus no comparison with the other group was possible. For this reason, it is not possible to rule out that these results are due to a test-retest effect. Finally, opposite to the results of the RCT, the CBCL score did not show significant changes in the MCNT 2.0 group. A possible interpretation of this result relates to differences in the specific difficulties and/or strengths of the children belonging to the two groups.

To summarize, children attending the MCNT 2.0 intervention improved their performances in all domains, especially in the motor domain whose improvement was more than one standard deviation. Notwithstanding, despite an increment in scores <1 standard deviation in the FSIQ and in the VABS II total score, these data are highly relevant from a clinical perspective for two main reasons. The first relates to the natural history of children with BIF and ACE that is not favorable, but is characterized by a high risk of long-term consequences. Thus, gaining a global improvement is potentially of great relevance. The second relates to the findings of a previous work from our group (33) in which results showed that working only on specific academic abilities produced improvement only in specific areas such as verbal memory and comprehension and did not generalize to adaptive and intellectual abilities. Moreover, this domain specific approach was associated with a paradoxical effect on behavior represented by a worsening on the CBCL scale. Consequently, our data support clinical decisions suggesting the importance of a multi domain therapeutic approach. To determine the long term clinical relevance of the MCNT intervention follow-up studies will be needed.

The present study is not free from limitations. This is a quasi-experimental study whose data from a non-randomized sample of subjects is compared to a secondary dataset from an RCT, according to the “Good Research Practices for Comparative Effectiveness Research” (34, 52, 66, 67). This approach potentially results in a selection bias of the children. To avoid this issue, we used the same inclusion criteria from the previous study and we included the first 20 consecutive children eligible for the study. Moreover, the children belonged to the same socioeconomic environment and from the same area of the children participating in the RCT. Moreover, the small group of participants prevent us from generalizing to the broad population of children with ACE and BIF. Finally, more follow-up is needed to evaluate the long-term effects of the treatment.

Despite these limitations, the herein data are relevant because of the importance to intervene in the developmental course of children experiencing ACE and with a BIF. This population is very vulnerable due to the high risk of school drop-out, poverty, and psychological problems in the adulthood (10, 13, 14, 68). Several data indicate that, in socio-economic disadvantaged contexts, children's IQ, together with the quality of the child/parent relationship, is one of the most important protective factor against maladaptive outcomes (36, 69). Indeed, one of the main goals of MCNT is to support resilience, through the multidomain and integrated approach that promotes the improvement of both the intellectual functioning and the emotional/relational competences of the children. The relational dimension is the real core of MCNT: everyone has an emotional and cognitive potential that can be enriched by the positive interaction with competent figures.

## Conclusions

To conclude, our data support the starting hypothesis of the positive effects of our intensive, multidomain approach in children with ACE and BIF, and that a body awareness and interlimbic coordination approach has a greater effect in improving the motor functioning. The treatment of neurodevelopmental disorders is often expensive due to the necessity of a long-term care (70, 71). Is not clear how protective factors and resilience work in modifying the association between ACE and psychopathology. On the basis of our experience, we believe that a multimodal approach intervening on the three major domains, the cognitive, the motor, and the emotion, may positively impact the developmental processes and thus help prevent maladaptive outcomes.

## Data Availability Statement

The datasets presented in this article are not readily available and will not be made publicly available because: the informed consent approved by the Ethics committee did not include any statement regarding the possibility to share the data. Requests to access the datasets should be directed to Gisella Baglio, gbaglio@dongnocchi.it.

## Ethics Statement

The studies involving human participants were reviewed and approved by the Ethics Committees of the Don Gnocchi Foundation and of the ASST S. Paolo and S. Carlo Hospital. Written informed consent to participate in this study was provided by the participants' legal guardian/next of kin.

## Author Contributions

GB, MZ, FB, and VB conceived the study and wrote the manuscript. GB, VB, MC, and MZ executed the study. SD helped with statistical analyses. All authors contributed to refinement of the manuscript and approved the final content.

## Conflict of Interest

The authors declare that the research was conducted in the absence of any commercial or financial relationships that could be construed as a potential conflict of interest.
